# Physical Activity and All-Cause Mortality by Age in 4 Multinational Megacohorts

**DOI:** 10.1001/jamanetworkopen.2024.46802

**Published:** 2024-11-21

**Authors:** David Martinez-Gomez, Mengyun Luo, Yu Huang, Fernando Rodríguez-Artalejo, Ulf Ekelund, Mercedes Sotos-Prieto, Ding Ding, Xiang-Qian Lao, Verónica Cabanas-Sánchez

**Affiliations:** 1 Department of Preventive Medicine and Public Health, School of Medicine, Universidad Autonoma de Madrid, Madrid, Spain; 2 CIBER of Epidemiology and Public Health, Madrid, Spain; 3 IMDEA-Food Institute, CEI UAM+CSIC, Madrid, Spain; 4 Prevention Research Collaboration, Sydney School of Public Health, The University of Sydney, Sydney, New South Wales, Australia; 5 Charles Perkins Centre, The University of Sydney, Sydney, New South Wales, Australia; 6 Department of Biomedical Sciences, City University of Hong Kong, Hong Kong, China; 7 Department of Sports Medicine, Norwegian School of Sports Sciences, Oslo, Norway; 8 Department of Chronic Diseases, Norwegian Institute of Public Health, Oslo, Norway; 9 Department of Environmental Health, Harvard T.H. Chan School of Public Health, Boston, Massachusetts

## Abstract

**Importance:**

Physical activity (PA) guidelines recommend the same amount of PA through adulthood to live longer.

**Objective:**

To explore whether there is an age-dependent association between PA and all-cause mortality and to investigate the age-dependent associations between other modifiable health factors (high educational level, not smoking, not regularly consuming alcohol, healthy body weight, and living without hypertension and diabetes) and mortality.

**Design, Setting, and Participants:**

This cohort study used a pooled analysis of 4 population-based prospective cohorts (National Health Interview Survey, 1997-2018; UK Biobank, 2006-2010; China Kadoorie Biobank, 2004-2008; and Mei Jau, 1997-2016). Data were analyzed from June 2022 to September 2024.

**Exposures:**

Self-reported leisure-time PA.

**Main Outcomes and Measures:**

The primary outcome was deaths identified through follow-up linkage to national death registries. Analyses were performed for the total sample and by age groups (20-29, 30-39, 40-49, 50-59, 60-69, 70-79, and ≥80 years). Cox proportional hazards regression models with stratification by study were used to calculate mortality hazard ratios and their 95% CIs for the pooled dataset and by age group.

**Results:**

A heterogeneous sample of 2 011 186 individuals (mean [SD] age, 49.1 [14.3] years; age range, 20-97 years; 1 105 581 women [55.0%]) were included. After a median (IQR) follow-up of 11.5 (9.3-13.5) years, 177 436 deaths occurred. The association between PA and mortality in the total sample showed a nonlinear dose-response pattern, but age modified this association (*P* for interaction <.001); PA was consistently associated with a lower risk of mortality across all age groups, but the reduction in risk was greater in older vs younger age groups, especially at high levels of PA. The hazard ratio for mortality associated with meeting the recommended PA in the total sample was 0.78 (95% CI, 0.77-0.79). This inverse association between meeting PA recommendations and mortality was somewhat greater as age increased (*P* for interaction <.001). Age also modified the associations of the other modifiable health factors with mortality (all *P* for interaction <.001), but the magnitude of associations was greater in younger vs older age groups.

**Conclusions and Relevance:**

In this pooled analysis of cohort studies, the association between PA and mortality risk remained consistent across the adult lifespan, which contrasts with other modifiable health factors, for which associations with mortality risk diminished with age. Given these findings, the promotion of regular PA is essential at all stages of adult life.

## Key Points

**Question:**

Does age modify the associations between physical activity and all-cause mortality?

**Findings:**

In this cohort study using a pooled analysis of 4 multicountry megacohorts including more than 2 million individuals aged 20 to 97 years, the beneficial association between meeting the physical activity recommendation (eg, 150 minutes per week of moderate-intensity physical activity) and mortality was greater as age increased. For other modifiable health factors, the associations were remarkably smaller as age increased.

**Meaning:**

The magnitude of the association between physical activity and mortality risk remains mainly consistent across the adult lifespan; therefore, promotion of physical activity is essential at all stages of adult life.

## Teaser

This cohort study examines whether there is an age-dependent association between physical activity and all-cause mortality at all stages of adult life.

## Introduction

Strong evidence suggests that meeting physical activity (PA) guidelines is associated with lower risk of death.^1^ However, it is unclear, but plausible, that this association may differ by age. For example, although PA levels tend to decline with aging,^2,3,4,5,6,7,8^ the absolute risk of death increases with age.^9,10,11,12^ Furthermore, the leading causes of death shift with age. A greater proportion of deaths among older adults are attributed to noncommunicable diseases, such as cancer and cardiovascular diseases, compared with younger adults, who have a greater proportion of deaths due to communicable diseases, injuries, accidents, and suicides.^9,10,11,12^ Despite these age-specific differences, PA guidelines recommend the same amount of PA for both adults and older adults: 150 to 300 minutes of moderate-intensity PA, 75 to 150 minutes of vigorous-intensity PA, or a combination of both for meaningful health benefits, even though lower amounts already offer some health benefits.^13^

Understanding the association between PA and mortality across age groups is vital for tailoring age-specific PA recommendations to optimize health benefits throughout the adult lifespan. Thus, we pooled individual participant data from 4 multicountry cohorts, including adults aged 20 to 97 years, to explore whether the magnitude and shape of the dose-response association between PA levels and mortality, as well as the association between meeting PA guidelines and mortality, is age dependent. In parallel, and to provide further context, we also investigated the age-dependent associations between other modifiable health factors, including educational level, smoking, alcohol consumption, body weight, hypertension, and diabetes, in the same pooled sample.

## Methods

### Study Design and Participants in the Pooled Analysis

We pooled data from 4 cohorts, each with more than half a million participants: the National Health Interview Survey (NHIS; 1997-2018) in the US,^14^ the UK Biobank (2006-2010) in the UK,^15^ the China Kadoorie Biobank (2004-2009) in China,^16^ and the Mei Jau cohort (MJ; 1997-2016) in Taiwan.^17^ Each cohort provides data accessible to external researchers, which includes detailed self-reported PA necessary to estimate energy expenditure (metabolic equivalents task [MET]–hours per week) and associated mortality records. Detailed descriptions of the study design and data collection procedures for these cohorts are available in previous publications.^18,19,20,21^ Procedures from these cohorts were approved by the US National Center for Health Statistics Research Ethics Review Board (NHIS), the North West Multi-Center Research Ethical Committee (UK Biobank), the China Center for Disease Control and Prevention and the Oxford Tropical Research Ethics Committee (China Kadoorie Biobank), and the Joint Chinese University of Hong Kong–New Territories East Cluster Clinical Research Ethics Committee and the National Cheng Kung University Research Ethics Committee (MJ). Participants in the 4 cohorts provided consent; it was not needed for the current work because the data are deidentified and publicly available, in accordance with 45 CFR §46. This report follows the Strengthening the Reporting of Observational Studies in Epidemiology (STROBE) reporting guideline. The analytical sample for the pooled analysis included participants with complete data on PA, mortality, and other study variables (eTable 1 in Supplement 1).

### Exposure Assessment

We focus our analyses on leisure-time PA to enhance the comparability of PA measures across studies and to enable the translation of our findings into actionable health promotion messages, consistent with previous pooled analyses.^13,22,23,24^ A detailed description of the PA measures and the harmonization process is presented in eTables 2 to 5 in Supplement 1. To calculate the total amount of baseline leisure-time PA in MET-hours per week, the MET value for the reported intensity was multiplied by the frequency and duration of PA. Participants were categorized as meeting the PA recommendations if they reported at least 7.5 MET-hours per week.^13^

### Mortality

Deaths in each cohort were identified through follow-up linkage to National Death registries. These registries include the US National Death Index for NHIS; National Health Service England and the National Health Service Central Register Scotland for UK Biobank; Chinese Centers for Disease Control and Prevention, National Health Insurance, and local street committees and village administrators for the China Kadoorie Biobank; and the Taiwan National Death File for MJ. The end of available follow-up data varied for each cohort: NHIS until December 31, 2019; UK Biobank until March 31, 2021; China Kadoorie Biobank until December 31, 2016; and MJ until May 31, 2019. Follow-up time was calculated from the date of the PA assessment to the date of death, lost to follow-up, or the end of follow-up in each study, whichever came first.

### Covariates

To ensure consistency across studies, we also harmonized covariates. Participants were categorized by the highest educational level attained into 3 groups: low (eg, primary education or lower), middle (eg, secondary education) and high (eg, university) education (eTable 6 in Supplement 1). Alcohol consumption was classified into 3 categories: never or occasional, infrequent (1-2 times per week), and regular (≥3 times per week) (eTable 7 in Supplement 1). Similarly, other covariates were harmonized, including smoking status (never, former, or current), and physician-diagnosed conditions, such as diabetes, hypertension, cardiovascular disease, and cancer, each categorized as no or yes. Body weight and height measurements followed standardized procedures, except in the NHIS, where these values were self-reported but were adjusted using the Stommel and Schoenborn equation.^25,26^ Body mass index (BMI) was calculated as weight in kilograms divided by height in meters squared. For the comparison analysis, we focused on the following 6 modifiable health factors derived from these covariates: high educational level, nonsmoking, infrequent or no alcohol consumption, and healthy body weight^27^ (defined as BMI ≥18.5 to <30.0 in the NHIS and UK Biobank, and ≥18.5 to <28.0 in the China Kadoorie Biobank and MJ), as well as living without hypertension and diabetes. These modifiable health factors have been identified as key factors associated with mortality according to previous studies of these and other cohorts.^28,29,30,31,32^

### Statistical Analysis

Descriptive statistics were presented as mean (SD), median (IQR), or number of participants (percentage). For the main analysis, we created 7 age groups: 20 to 29, 30 to 39, 40 to 49, 50 to 59, 60 to 69, 70 to 79, and 80 or more years. In this study, *older adults* refers to participants aged 60 years and older.^33,34^ Cox proportional hazards regression models with stratification by study were used to calculate mortality hazard ratios (HRs) and their 95% CIs for the pooled dataset and by age group. The proportional hazards assumptions were tested, with no evidence of nonproportionality detected.

Initially, we examined the dose-response association between PA and all-cause mortality across the range of MET-hours per week values. This was done using restricted cubic splines with knots at 10th, 50th, and 90th percentiles of PA, adjusting for age (years), sex (men or women), educational level (low, middle, or high), alcohol consumption (never or occasional, infrequent, or regular), smoking (never, former, or current), BMI (<18.5, 18.5-22.9, 23.0-26.9, 27.0-27.9, 28.0-29.9, 30.0-34.9, or ≥35.0), diabetes (no or yes), hypertension (no or yes), cardiovascular disease (no or yes), and cancer (no or yes). We subsequently examined the association between meeting the recommended amount of PA and all-cause mortality, adjusting for age, sex, cardiovascular disease, cancer, and the 6 modifiable health factors (no or yes), with a single multiplicative interaction term incorporated in the model to assess whether the associations of PA with mortality varied across different age groups using a likelihood ratio test.

For the comparison analysis, we initially used Cox regression to individually examine the associations between each of the 6 modifiable health factors and all-cause mortality. This analysis adjusted for age, sex, cardiovascular diseases, cancer, whether the recommended PA levels were met (yes or no), and the remaining 5 modifiable health factors (categorized as yes or no for each). Previously, odds ratios (95% CI) were calculated to examine the association between meeting the recommended PA and the other health factors.

In sensitivity analyses, and to account for differences across cohorts, we created separate cohort-specific models. Main analyses were also stratified by sex and region (Western or Asian). Sensitivity analyses were performed to minimize reverse causation and rule out preexisting subclinical disease or worse health status at baseline, so that we repeated the analysis after excluding (1) former or current smokers (777 569 individuals), (2) those with prevalent chronic conditions such as cardiovascular disease and cancer (190 781 individuals), and (3) those who died in the first 2 years of follow-up (10 826 individuals). An additional analysis involved examining the main associations with 5-year mortality; this was to confirm robustness at short-term follow-up, particularly in age groups, such as young adults, with low mortality rates. All analyses were performed using Stata statistical software version 16.1 (StataCorp), and all *P* values were 2-sided with a significance level of .05. Data were analyzed from June 2022 to September 2024.

## Results

**Figure 1.**
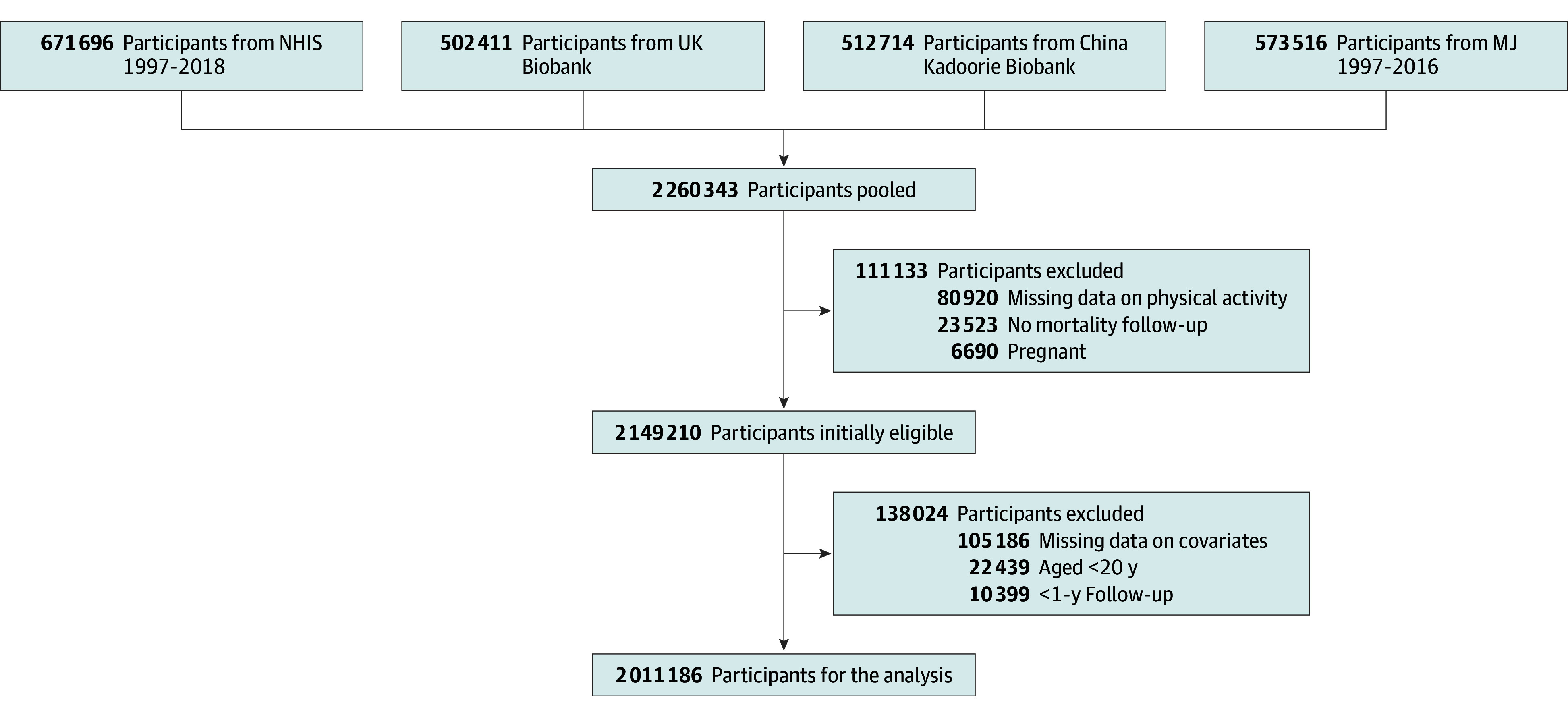
Flowchart of Participant Selection MJ indicates Mei Jau; and NHIS, National Health Interview Survey.

In the pooled sample of 2 011 186 individuals (mean [SD] age, 49.1 [14.3] range, 20-97 years; 1 105 581 women [55.0%]) (Figure 1), we observed 177 436 deaths (8.8%) over a median (IQR) follow-up of 11.5 (9.3-13.5) years (Table). There was substantial heterogeneity in baseline characteristics between cohorts, although more similarities were observed by region.

**Table.** Characteristics of Participants in the Pooled Total Sample and by Cohort

Characteristic	Participants, No. (%)				
	Total sample (N = 2 011 186)	NHIS 1997-2018 (n = 533 439)	UK Biobank (n = 481 615)	CK Biobank (n = 510 419)	MJ 1997-2016 (n = 485 713)
Sex					
Female	1 105 581 (55.0)	292 417 (54.8)	262 444 (54.5)	301 639 (59.1)	249 081 (51.3)
Male	905 605 (45.0)	241 022 (45.2)	219 171 (45.5)	208 780 (40.9)	236 632 (48.7)
Age group, y					
20-29	207 726 (10.3)	88 060 (16.5)	0	0	119 666 (24.6)
30-39	345 857 (17.2)	101 273 (19.1)	5 (0.1)	77 533 (15.2)	167 046 (34.4)
40-49	450 221 (22.4)	98 865 (18.5)	113 246 (23.5)	152 497 (29.9)	85 613 (17.6)
50-59	471 148 (23.4)	90 683 (17.0)	160 660 (33.3)	157 047 (30.8)	62 758 (12.9)
60-69	408 413 (20.3)	74 329 (13.9)	205 408 (42.7)	90 935 (17.8)	37 741 (7.8)
70-79	97 497 (4.9)	51 352 (9.6)	2296 (0.4)	32 407 (6.3)	11 442 (2.4)
≥80	30 324 (1.5)	28 877 (5.4)	0	0	1447 (0.3)
Educational level					
Low (primary education or lower)	524 467 (26.1)	87 238 (16.4)	81 590 (16.9)	258 677 (50.7)	96 962 (20.0)
Middle (secondary education)	949 278 (47.2)	281 250 (52.7)	241 258 (50.1)	221 825 (43.5)	204 945 (42.2)
High (university)	537 441 (26.7)	164 951 (30.9)	158 767 (33.0)	29 917 (5.9)	183 806 (37.8)
Smoking					
Never	1 233 617 (61.3)	302 317 (56.7)	264 323 (54.9)	316 538 (62.0)	350 439 (72.2)
Former	350 164 (17.4)	123 002 (23.1)	167 194 (34.7)	30 190 (5.9)	29 778 (6.1)
Current	427 405 (21.3)	108 120 (20.3)	50 098 (10.4)	163 691 (32.1)	105 496 (21.7)
Alcohol consumption					
Never or occasional	1 344 119 (66.8)	354 059 (66.4)	146 029 (30.3)	434 588 (85.2)	409 443 (84.3)
Infrequent	301 695 (15.0)	111 383 (20.9)	124 657 (25.9)	15 963 (3.1)	49 692 (10.2)
Regular	365 372 (18.2)	67 997 (12.8)	210 929 (43.8)	59 868 (11.7)	26 578 (5.5)
Physical activity, median (IQR), MET-h/wk	3.5 (0.0-15.8)	6.8 (0.0-23.2)	11.4 (3.7-25.0)	0	2.3 (0.0-8.8)
Meeting the recommendation^a^	786 682 (39.1)	255 645 (47.9)	298 376 (62.9)	97 065 (19.0)	135 596 (27.9)
Body mass index, mean (SD)^b^	25.5 (4.9)	27.8 (5.4)	27.4 (4.8)	23.6 (3.3)	23.0 (3.6)
Healthy body weight^c^	1 558 410 (77.5)	362 298 (67.9)	362 609 (75.3)	434 643 (85.2)	398 860 (82.1)
Hypertension	386 239 (19.2)	162 322 (30.4)	129 220 (26.8)	59 180 (11.6)	35 517 (7.3)
Diabetes	98 706 (4.9)	46 152 (8.7)	24 597 (5.1)	15 988 (3.1)	11 969 (2.5)
Cardiovascular disease	112 730 (5.6)	47 166 (8.8)	27 042 (5.6)	23 665 (4.6)	14 857 (3.1)
Cancer	90 359 (4.5)	45 806 (8.6)	36 890 (7.7)	2468 (0.5)	5195 (1.1)
Follow-up duration, median (IQR), y	11.5 (9.3-13.5)	9.8 (5.4-16.0)	12.1 (11.4-12.8)	10.1 (9.2-11.1)	15.5 (10.9-19.6)
Deaths	177 436 (8.8)	74 058 (13.9)	31 681 (6.6)	41 833 (8.2)	29 864 (6.1)

Abbreviations: CK, China Kadoorie; MET, metabolic equivalents task; MJ, Mei Jau; NHIS, National Health Interview Survey.

^a^ Refers to doing at least 7.5 MET-hours per week.

^b^ Body mass index is calculated as weight in kilograms divided by height in meters squared.

^c^ Defined as body mass index greater than or equal to 18.5 to less than 30.0 in the NHIS and UK Biobank, and greater than or equal to 18.5 to less than 28.0 in the China Kadoorie Biobank and MJ.

**Figure 2.**
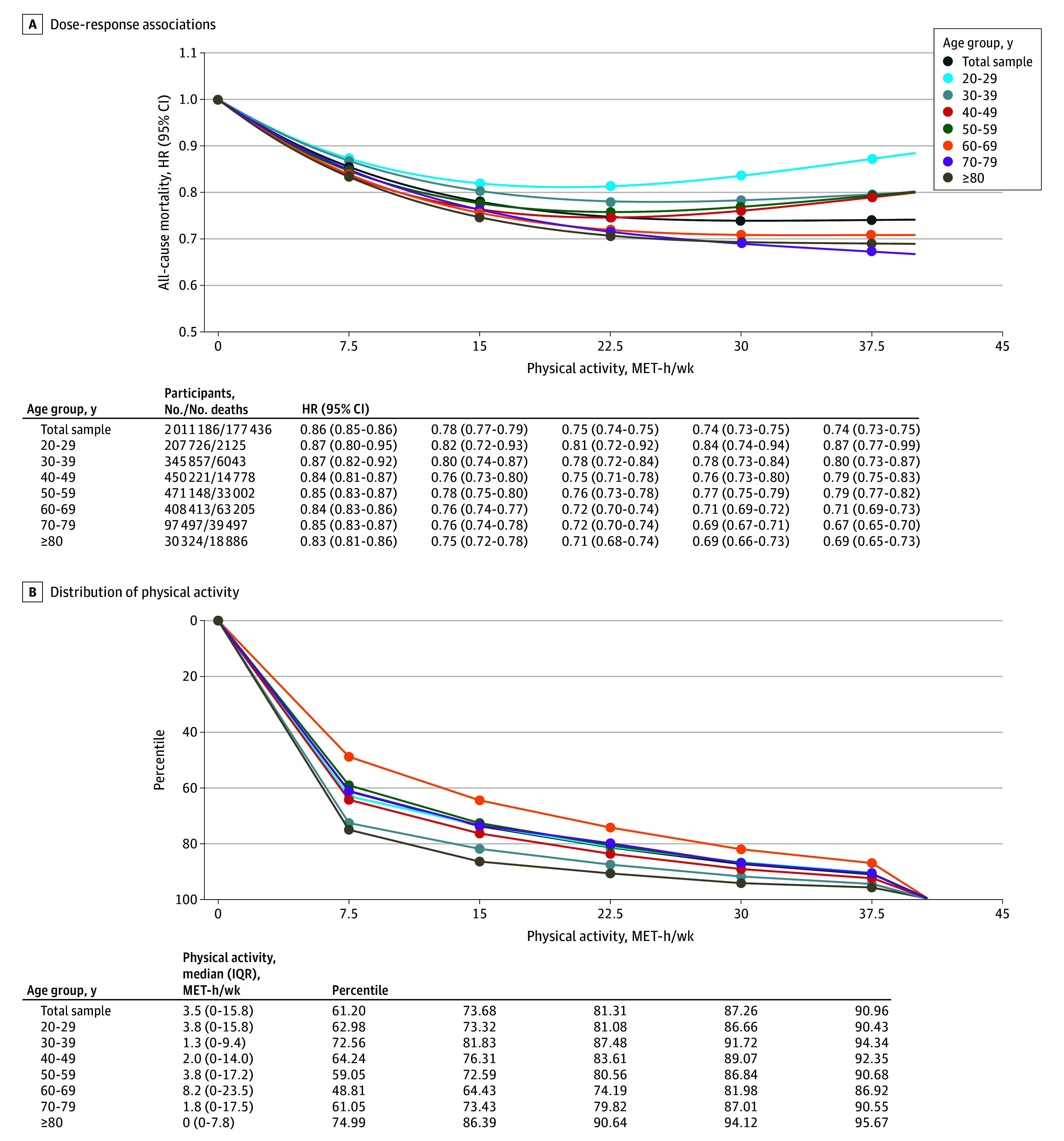
Dose-Response Association Between Physical Activity and All-Cause Mortality and Distribution of Physical Activity Levels in the Total Pooled Sample and by Age Group Values of 7.5, 15.0, 22.5, 30.0, and 37.5 metabolic equivalents task hours per week (MET-h/wk) are equivalent to 1, 2, 3, 4, and 5 times the recommended amount of physical activity, respectively. The reference was 0 MET-h/wk in both graphs. Physical activity was truncated to 40 MET-h/wk. Analyses were adjusted for study, age, sex, educational level, alcohol consumption, smoking, body mass index, diabetes, hypertension, cardiovascular disease, and cancer. *P* for age-group interaction <.001. HR indicates hazard ratio.

In the total sample, the association between PA and all-cause mortality showed a nonlinear dose-response curve (Figure 2 and eFigure 1 in Supplement 1). Compared with the inactive referent group (0 MET-hours per week), engaging in 7.5 MET-hours per week (the recommended level of PA) was associated with a 14% lower mortality risk, 15.0 MET-hours per week (twice the recommended level) was associated with 22% lower risk, 22.5 MET-hours per week (3 times the recommended level) was associated with 25% lower risk, 30 MET-hours per week (4 times the recommended level) was associated with 26% lower risk, and 37.5 MET-hours per week (5 times the recommended level) was associated with 26% lower risk. Lower amounts of PA also were associated with significantly lower mortality risks (eg, half of the recommended amount [3.75 MET-hours per week] was associated with an 8% lower mortality risk). Age was a modifier of the association between PA and mortality (*P* for interaction <.001), indicating that although PA was consistently associated with a lower risk of mortality across all age groups, the magnitude of risk reduction was more pronounced in older adults than in younger ones, particularly at higher levels of PA. The greatest reduction in risk was observed at levels of PA approximately 4 to 5 times higher than current recommendations (around 22.5-30.0 MET-hours per week; 81.3-87.3rd percentile) for older adults, in contrast to twice the recommendation level (around 15 MET-hours per week; 73.7th percentile) for younger participants (Figure 2 and eFigure 2 in Supplement 1). Of note, only 25% of adults participated in PA exceeding the 15 MET-hours per week threshold.

**Figure 3.**
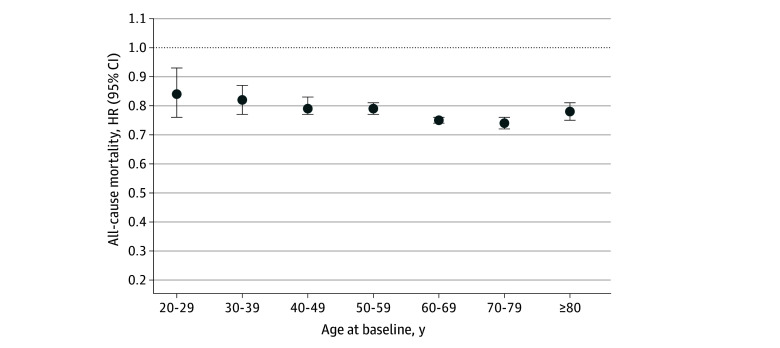
Association Between Meeting the Recommended Physical Activity and All-Cause Mortality in the Pooled Sample by Age Group Meeting the physical activity recommendation is defined as doing at least 7.5 metabolic equivalents task hours per week. Analyses were adjusted for study, age, sex, cardiovascular disease, cancer, and other health factors (including high educational level, not smoking, not regularly consuming alcohol, healthy body weight, living without hypertension, and living without diabetes). *P* for age-group interaction <.001. HR indicates hazard ratio.

The HR for mortality associated with meeting the recommended PA (vs not met) in the total sample was 0.78 (95% CI, 0.77-0.79). When stratified by age group (*P* for interaction <.001), the inverse association between adherence to PA recommendations and mortality became somewhat more pronounced with increasing age (Figure 3). Specifically, the HR was 0.84 (95% CI, 0.76-0.93) for the 20 to 29 years group, and improved to 0.78 (95% CI, 0.75-0.81) for those in the 80 years or older group. Analyses specific to each cohort, as well as those stratified by sex and region, yielded similar results (eTables 8-10 in Supplement 1).

**Figure 4.**
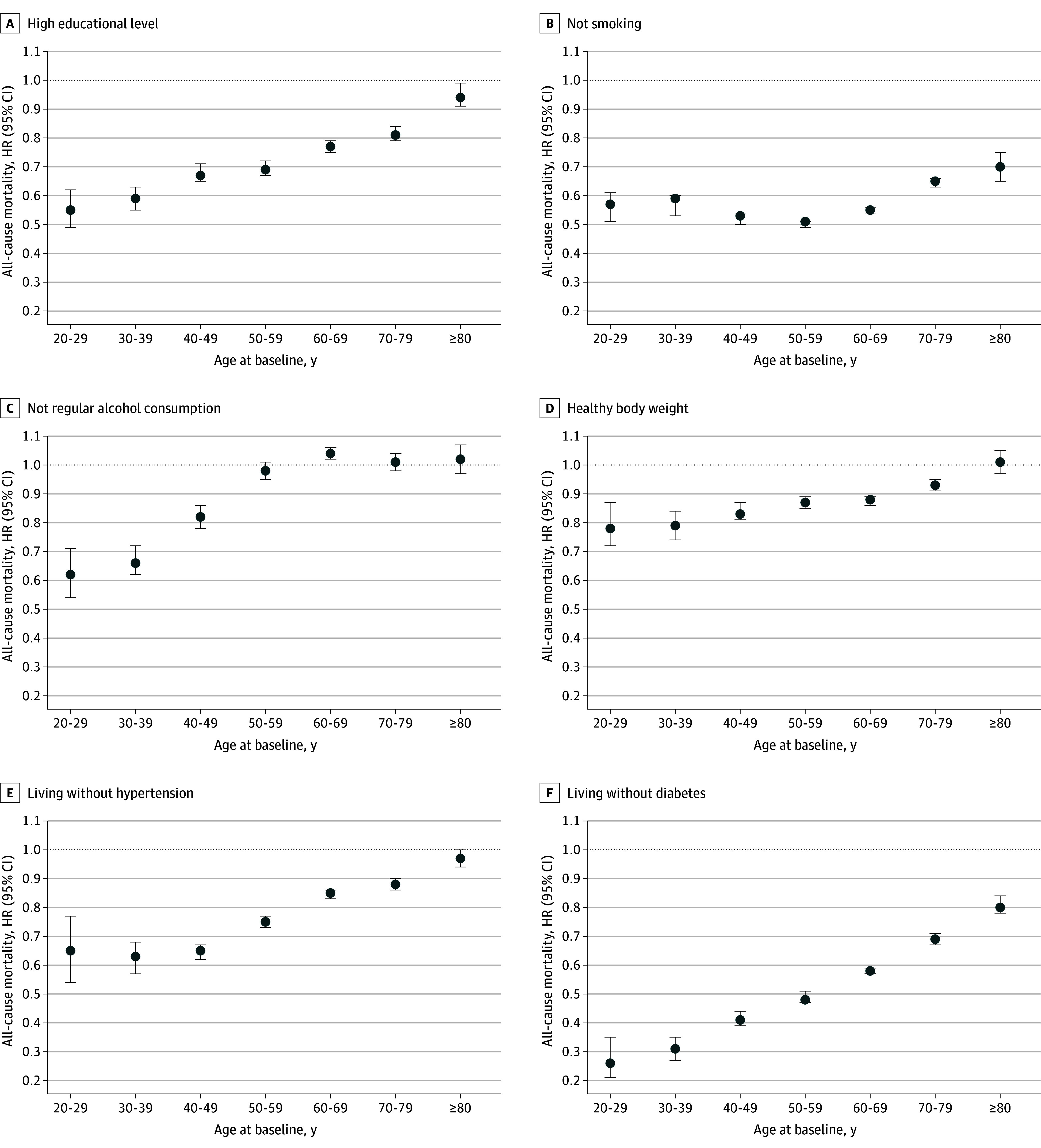
Association Between 6 Health Factors and All-Cause Mortality in the Pooled Sample by Age Group Healthy body weight was defined as body mass index (calculated as weight in kilograms divided by height in meters squared) greater than or equal to 18.5 to less than 30.0 in the National Health Interview Survey and UK Biobank, and body mass index greater than or equal to 18.5 to less than 28.0 in the China Kadoorie Biobank and Mei Jau cohort. Analyses were adjusted for study, age, sex, cardiovascular disease, cancer, recommended physical activity, and the health factors showed in the figure. All *P* for age-group interaction <.001. When not smoking was defined as never smoking (vs current or former smoking), the hazard ratios (HRs) were 0.66 (95% CI, 0.66-0.67) for the total sample, and 0.61 (95% CI, 0.56-0.67) for participants aged 20 to 29 years, 0.64 (95% CI, 0.61-0.68) for those aged 30 to 39 years, 0.63 (95% CI, 0.61-0.65) for those aged 40 to 49 years, 0.60 (95% CI, 0.59-0.62) for those aged 50 to 59 years, 0.65 (95% CI, 0.64-0.66) for those aged 60 to 69 years, 0.69 (95% CI, 0.67-0.70) for those aged 70 to 79 years, and 0.81 (95% CI, 0.78-0.83) those aged 80 years and older.

Meeting the recommended PA was positively associated with 5 modifiable health factors, and was inversely associated with not regularly consuming alcohol (eTable 11 in Supplement 1). In the total sample, the HRs for mortality were 0.74 (95% CI, 0.73-0.75) for high educational level, 0.55 (95% CI, 0.54-0.56) for not smoking, 0.99 (95% CI, 0.98-1.01) for not regularly consuming alcohol, 0.88 (95% CI, 0.87-0.89) for healthy body weight, 0.83 (95% CI, 0.82-0.84) for living without hypertension, and 0.61 (95% CI, 0.60-0.62) for living without diabetes (Figure 4). Age also modified the associations between the 6 modifiable health factors and all-cause mortality (all *P* for interaction <.001). However, the magnitude of associations between these health factors and mortality risk was more pronounced in younger age groups compared with older ones.

Sensitivity analyses conducted on subgroups, such as never smokers, individuals without prevalent chronic conditions, and those dying in the first 2 years of follow-up, showed identical results (eTable 12 in Supplement 1). Furthermore, these analyses confirmed an age-dependent association between PA and mortality even during short-term follow-up.

## Discussion

In this cohort study with a pooled analysis of more than 2 million adults, we observed that age somewhat modifies the association between meeting the PA recommendations and all-cause mortality. This age-dependent association showed a distinct pattern compared with those observed for other modifiable health factors. Although the mortality risk reduction associated with meeting the PA recommendations either remained stable or slightly increased with age, the benefits related to other health factors diminished as age advanced.

Overall, previous evidence^35,36,37,38,39,40,41^ indicates that the impact of certain modifiable health factors on mortality risk diminishes with age, indicating that their relative importance is lower among older adults compared with younger individuals. This observation could be attributed to selection bias, suggesting that individuals who are biologically more vulnerable to the adverse effects of risk factors may die earlier, leaving a population of older adults who are inherently less susceptible (ie, survivors), thereby decreasing the apparent association between these risk factors and mortality with advancing age. Conversely, extensive research within prospective cohorts that include a large proportion of older adults has consistently highlighted PA as a crucial determinant for enhancing survival later in life.^23,42^ Furthermore, stratified analyses from these studies have revealed age-dependent associations between PA and mortality. For example, Arem et al^23^ pooled data from 6 Western cohorts (5 from the US and 1 from Sweden) as part of the National Cancer Institute Cohort Consortium, encompassing 661 137 men and women with median age of 62 years (range, 21-98 years), and identified a significant interaction (*P* < .001) across 4 age groups (ie, <50, 50-59, 60-69, and ≥70 years). Similarly, Liu et al^42^ analyzed data from 467 729 adults across 9 Asian cohorts within the Asia Cohort Consortium, with a mean age of 55 years (range, 48-60 years), and observed that the association between PA and mortality was more pronounced among older participants (≥65 years) compared with younger ones (<55 years and 55-64 years) at baseline (*P* for interaction = .04).

Differences in the association between PA, as measured in MET-hours per week, and mortality risk became notably more pronounced between younger and older age groups, particularly beyond the 15 MET-hours per week threshold but taking into account that any amount of PA was better than none. Yet, on average, only 25% of adults participate in PA exceeding this level, with engagement in such activities sharply declining from the age of 60 years onward. Consequently, if a larger fraction of older adults were engaged in PA levels beyond 15 MET-hours per week, a more substantial reduction in mortality risk could potentially be observed. Several factors contribute to why the mortality benefits of PA may be similar or even greater for older compared with younger adults. First, PA is more associated with certain causes of death,^1,13^ mainly those affecting the circulatory system,^43,44^ and heart disease remains the leading cause of death in the elderly.^9,10,11,12^ Second, aging is accompanied by a decline in task performance, mobility, fitness levels, coordination, and exercise economy, suggesting that older adults may reap substantial benefits from PA at lower levels of intensity owing to their reduced capacity for physical exertion.^45,46^ Third, ample evidence supports PA’s role in mitigating major aging hallmarks, such as genomic instability and mitochondrial dysfunction, thereby underscoring its preventative potential against the physiological processes of aging.^47^ Fourth, PA is instrumental in slowing the progression of functional impairments and frailty, which are critical factors associated with unhealthy aging and increased mortality risk, by counteracting the decline in physiological reserve and heightened vulnerability to stressors seen in old age. However, the greater association observed in older age groups might also reflect the capacity for doing PA (often considered a vital sign of health at advanced ages), with the somewhat increased association possibly attributable to more residual confounding by health status.^33^

Global and other PA guidelines do not differentiate recommendations by age; the advised amounts of PA for younger, middle-aged, and older adults are uniformly the same.^13^ Systematic reviews underpinning these recommendations have consistently demonstrated that meeting these PA levels is associated with a 20% to 30% reduction in mortality risk compared with individuals who do not meet these criteria. Our study introduces new insights, further affirming that the mortality benefits associated with PA not only persist across different age groups but may also slightly enhance with age. From a public health viewpoint, it is crucial to communicate to adults that engaging in an adequate amount of PA remains critically important throughout the lifespan, gaining even greater importance as one ages. Policy actions must be addressed to facilitate and promote desired amounts of PA that can promote PA engagement and sustainability at all stages of adult life. Our results also lend support for the current PA guidelines where adults of all ages are recommended the same amount of PA.

### Limitations and Strengths

Several limitations warrant mention. PA was self-reported, introducing potential recall and social desirability biases, such as the tendency to overreport, which may skew the associations toward the null.^48^ Also, we only examined PA during leisure and, therefore, could not evaluate the age-dependent associations with other domains (eg, occupation, household, and transportation) of PA. Similarly, PA assessments in this work did not allow us to examine associations by modes of PA, which may also contribute to different outcomes by age, and this warrants consideration in future studies. Furthermore, PA was assessed at a single time point, neither accounting for changes over the follow-up period nor information on PA history before the time point. This limitation potentially biases our results toward the null but also prevents suggesting potential key interventions to promote PA varying by age. On the other hand, certain modifiable health factors were also self-reported, necessitating cautious interpretation of these findings until further validated by studies with more precise measurement techniques, especially for biological factors (eg, through blood pressure monitoring, fasting glucose, or glycosylated hemoglobin tests) and alcohol consumption (eg, repeated measures of 24-hour dietary recall or dietary histories). Despite this limitation, the observed age-dependent patterns of association with mortality align with those found in previous studies using more accurate methods.^35,36,37,38,39,40,41^ While numerous sensitivity analyses were conducted, the potential for reverse causation (eg, age-related morbidity leading to decreased PA levels) and selection bias (eg, individuals more susceptible to risk factors dying younger) remains a consideration not just for PA but for the modifiable health factors across the lifespan. Although adjustments were made for a wide array of covariates, the risk of residual confounding due to unmeasured (eg, diet and medication) or imprecisely measured (eg, socioeconomic status, self-reported weight and height, and alcohol consumption) factors cannot be entirely ruled out. Furthermore, because this was an observational study, causality cannot be definitively established.

## Conclusions

In this pooled analysis of cohort studies, the association between PA and mortality risk remained consistent across the adult lifespan. This contrasts with other modifiable health factors, including educational level, smoking, alcohol consumption, body weight, hypertension, and diabetes, where we observed that their associations with mortality risk diminished with age. Given these findings, the promotion of regular PA is essential at all stages of adult life.

## Notes

Supplement 1. **eTable 1.** Baseline differences between participants included and excluded in the analyses **eTable 2.** Description of leisure-time physical activity questionnaires: NHIS 1997-2018 **eTable 3.** Description of leisure-time physical activity questionnaires: UK Biobank **eTable 4.** Description of leisure-time physical activity questionnaires: China Kadoorie Biobank **eTable 5.** Description of leisure-time physical activity questionnaires: MJ Taiwan 1997-2016 **eTable 6.** Harmonization of the educational level for the pooled analysis **eTable 7.** Harmonization of alcohol consumption for the pooled analysis **eFigure 1.** Dose-response association between physical activity and all-cause mortality in the pooled sample and by age group **eFigure 2.** Dose-response association between physical activity and all-cause mortality in the pooled sample by age group **eTable 8.** Risk of mortality associated with meeting the recommended physical activity, stratified by cohort **eTable 9.** Risk of mortality associated with meeting the recommended physical activity excluding one cohort at a time **eTable 10.** Risk of mortality associated with meeting the recommended physical activity in the pooled four cohorts and by age group stratified by sex and region subgroups **eTable 11.** Associations between meeting the recommended physical activity and other modifiable health behaviors **eTable 12.** Mortality associated with meeting the recommended physical activity in the pooled four cohorts and by age group when excluding some participants and when considering mortality at 5-years follow-up

Supplement 2.
Data Sharing Statement
