# Misspelled Author’s Name

**DOI:** 10.1001/jamanetworkopen.2024.60155

**Published:** 2025-01-15

In the Original Investigation titled “Physical Activity and All-Cause Mortality by Age in 4 Multinational Megacohorts,”^1^ published November 21, 2024, coauthor Xiang-Qian Lao’s name was misspelled. This article has been corrected.^1^
