# Supplementary material for: Physical Activity and All-Cause Mortality by Age in 4 Multinational Megacohorts
Source: JAMA Netw Open. 2024 Nov 21;7(11):e2446802. doi: 10.1001/jamanetworkopen.2024.46802 (PMC11582934; doi:10.1001/jamanetworkopen.2024.46802)
Supplement: Supplement 1. — eTable 1. Baseline differences between participants included and excluded in the analyses eTable 2. Description of leisure-time physical activity questionnaires: NHIS 1997-2018 eTable 3. Description of leisure-time physical activity questionnaires: UK Biobank eTable 4. Description of leisure-time physical activity questionnaires: China Kadoorie Biobank eTable 5. Description of leisure-time physical activity questionnaires: MJ Taiwan 1997-2016 eTable 6. Harmonization of the educational level for the pooled analysis eTable 7. Harmonization of alcohol consumption for the pooled analysis eFigure 1. Dose-response association between physical activity and all-cause mortality in the pooled sample and by age group eFigure 2. Dose-response association between physical activity and all-cause mortality in the pooled sample by age group eTable 8. Risk of mortality associated with meeting the recommended physical activity, stratified by cohort eTable 9. Risk of mortality associated with meeting the recommended physical activity excluding one cohort at a time eTable 10. Risk of mortality associated with meeting the recommended physical activity in the pooled four cohorts and by age group stratified by sex and region subgroups eTable 11. Associations between meeting the recommended physical activity and other modifiable health behaviors eTable 12. Mortality associated with meeting the recommended physical activity in the pooled four cohorts and by age group when excluding some participants and when considering mortality at 5-years follow-up [file jamanetwopen-e2446802-s001.pdf]

Supplemental Online Content

Martinez-Gomez D, Luo M, Huang Y, et al. Physical activity and all-cause mortality by age in 4 multinational megacohorts. *JAMA Netw Open*. 2024;7(11):e2446802. doi:10.1001/jamanetworkopen.2024.46802

eTable 1. Baseline differences between participants included and excluded in the analyses
eTable 2. Description of leisure-time physical activity questionnaires: NHIS 1997-2018
eTable 3. Description of leisure-time physical activity questionnaires: UK Biobank
eTable 4. Description of leisure-time physical activity questionnaires: China Kadoorie Biobank
eTable 5. Description of leisure-time physical activity questionnaires: MJ Taiwan 1997-2016
eTable 6. Harmonization of the educational level for the pooled analysis
eTable 7. Harmonization of alcohol consumption for the pooled analysis
eFigure 1. Dose-response association between physical activity and all-cause mortality in the pooled sample and by age group
eFigure 2. Dose-response association between physical activity and all-cause mortality in the pooled sample by age group
eTable 8. Risk of mortality associated with meeting the recommended physical activity, stratified by cohort
eTable 9. Risk of mortality associated with meeting the recommended physical activity excluding one cohort at a time
eTable 10. Risk of mortality associated with meeting the recommended physical activity in the pooled four cohorts and by age group stratified by sex and region subgroups
eTable 11. Associations between meeting the recommended physical activity and other modifiable health behaviors
eTable 12. Mortality associated with meeting the recommended physical activity in the pooled four cohorts and by age group when excluding some participants and when considering mortality at 5-years follow-up

This supplemental material has been provided by the authors to give readers additional information about their work.

© 2024 Martinez-Gomez D et al. *JAMA Network Open.*

eTable 1. Baseline differences between participants included and excluded in the analyses

	**Participants for the analysis**	**Participants excluded for the analysis**
Number of participants	2011186	249157
Sex		
Women, n (%)	1105581 (55.0)	143913 (57.7)
Men, n (%)	905605 (45.0)	105244 (42.2)
Age groups, n (%)		
< 20 years	0 (0)	24264 (9.7)
20-29 years	207726 (10.3)	38527 (15.5)
30-39 years	345857 (17.2)	48122 (19.3)
40-49 years	450221 (22.4)	42520 (17.1)
50-59 years	471148 (23.4)	38620 (15.5)
60-69 years	408413 (20.3)	33322 (13.4)
70-79 years	97497 (4.9)	14526 (5.8)
80+ years	30324 (1.5)	9256 (3.7)
Study		
NHIS 1997-2018	533439 (26.5)	138257 (55.5)
UK Biobank	481615 (24.0)	20802 (8.3)
CK Biobank	510419 (25.4)	2295 (0.9)
MJ 1997-2016	485713 (24.1)	87803 (35.2)

© 2024 Martinez-Gomez D et al. *JAMA Network Open.*

eTable 2. Description of leisure-time physical activity questionnaires: NHIS 1997-2018

NHIS 1997-2018				
**Questions and original response options**	**Recoded response options**	**Intermediate variables calculated**	**Energy cost assigned for harmonization**	**Total leisure-time PA (METs-h/week)**
**Question (Q)1.1.** How often do you do light or moderate activities for at least 10 minutes that cause only light sweating or a slight to moderate increase in breathing or heart rate?		Frequency in MPA (times/week) =number of time units * time units<br><br>Duration in MPA (h/time)= number of time units * time units<br><br>Total time in MPA= frequency * duration (h/week)	3.5 METs	Total LTPA= MPA (h/week) * 3.5 (METs) + 2 * VPA (h/week) * 7.5METs
Number of time units<br>Never<br>1-999 times<br>Unable to do this type of activity<br>Time Units<br>Never<br>Per day<br>Per week<br>Per month<br>Per year	Number of time units<br>0 times<br>1-999 times<br>0 times<br>Time Units<br>0 per week<br>7 per week<br>1 per week<br>1/4 per week<br>1/52.143 per week			
**Q1.2.** About how long do you do these light or moderate leisure-time physical activities each time?				
Number Time Units<br>1-995<br>Time Units<br>Minutes<br>Hours	Number Time Units<br>1-995<br>Time Units<br>1/60 hour<br>1 hour			
**Q2.1.** How often do you do vigorous activities for at least 10 minutes that cause heavy sweating or large increases in breathing or heart rate?		Frequency in VPA (times/week) =number of time units * time units<br><br>Duration in VPA (h/time) = number of time units * time units<br><br>Total time in VPA= frequency * duration (h/week)	7.5 METs	
Number of time units<br>Never<br>1-999 times<br>Unable to do this type of activity<br>Time Units<br>Never<br>Per day<br>Per week<br>Per month<br>Per year	Number of time units<br>0 times<br>1-999 times<br>0 times<br>Time Units<br>0 per week<br>7 per week<br>1 per week<br>1/4 per week<br>1/52.143 per week			
**Q2.2.** About how long do you do these vigorous leisure-time physical activities each time?				
Number Time Units<br>1-995<br>Time Units<br>Minutes<br>Hours	Number Time Units<br>1-995<br>Time Units<br>1/60 hour<br>1 hour			
**Example of calculation**: participant with the following responses:<br>Q1.1: 3 times per week; Q1.2: 30 min; Q2.1: 6 times per month; Q2.2: 45 min.<br>LTPA = 3 times/week * 0.5h/time * 3.5 METs + 2 * 1.5 times/week * 0.75 h/time * 7.5 METs = 5.25 METs-h/week + 16.875 METs-h/week<br>LTPA= 22.125 METS-h/week				
**Example reference**: doi: 10.1016/ j.diabet.2022.101410				

© 2024 Martinez-Gomez D et al. *JAMA Network Open.*

eTable 3. Description of leisure-time physical activity questionnaires: UK Biobank

UK Biobank				
**Questions and original response options**	**Recoded response options**	**Intermediate variables calculated**	**Energy cost assigned for harmonization**	**Total leisure-time PA (METs-h/week)**
**Question (Q)1.** In the last 4 weeks did you spend any time doing the following? (You can select more than one answer)				
Walking for pleasure (not as a means of transport)				
Light DIY (e.g.: pruning, watering the lawn)				
Heavy DIY (e.g.: weeding, lawn mowing, carpentry, digging)				
Strenuous sports				
Other exercises (e.g.: swimming, cycling, keep fit)				
None of the above				
**Q2.1.** How many times in the last 4 weeks did you go walking for pleasure?		Total time in walking (h/week) = frequency in walking (times/week) * duration in walking (h/time)	3.5 METs	Total LTPA= Walking (h/week) * 3.5 METs + light DIY (h/week) * 2.5 METs + heavy DIY (h/week) * 5.5 METs + strenuous sports (h/week) * 7.5 METs + other exercises (h/week) * 5.5 METs
None (if not selected in question 1)	0 times/week			
Once in the last 4 weeks	0.25 times/week			
2-3 times in the last 4 weeks	0.625 times/week			
Once a week	1 times/week			
2-3 times a week	2.5 times/week			
4-5 times a week	4.5 times/week			
Every day	7 times/week			
**Q2.2.** Each time you went walking for pleasure, about how long did you spend doing it?				
None (if not selected in question 1)	0 h			
Less than 15 minutes	0.125 h (7.5 min)			
Between 15 and 30 minutes	0.375 h (22.5 min)			
Between 30 minutes and 1 hour	0.75 h (45 min)			
Between 1 and 1.5 hours	1.25 h			
Between 1.5 and 2 hours	1.75 h			
Between 2 and 3 hours	2.5 h			
Over 3 hours	3.5 h			
**Q3.1.** How many times in the last 4 weeks did you do light DIY?		Total time in light DIY (h/week) = frequency in light DIY (times/week) * duration in light DIY(h/time)	2.5 METs	
None (if not selected in question 1)	0 times/week			
Once in the last 4 weeks	0.25 times/week			
2-3 times in the last 4 weeks	0.625 times/week			
Once a week	1 times/week			
2-3 times a week	2.5 times/week			
4-5 times a week	4.5 times/week			
Every day	7 times/week			
**Q3.2.** Each time you did light DIY, about how long did you spend doing it?				
None (if not selected in question 1)	0 h			
Less than 15 minutes	0.125 h (7.5 min)			
Between 15 and 30 minutes	0.375 h (22.5 min)			
Between 30 minutes and 1 hour	0.75 h (45 min)			
Between 1 and 1.5 hours	1.25 h			
Between 1.5 and 2 hours	1.75 h			
Between 2 and 3 hours	2.5 h			
Over 3 hours	3.5 h			
**Q4.1.** How many times in the last 4 weeks did you do heavy DIY?		Total time in heavy DIY (h/week) = frequency in heavy DIY (times/week) * duration in heavy DIY (h/time)	5.5 METs	
None (if not selected in question 1)	0 times/week			
Once in the last 4 weeks	0.25 times/week			
2-3 times in the last 4 weeks	0.625 times/week			
Once a week	1 times/week			
2-3 times a week	2.5 times/week			
4-5 times a week	4.5 times/week			
Every day	7 times/week			
**Q4.2.** Each time you did heavy DIY, about how long did you spend doing it?				
None (if not selected in question 1)	0 h			
Less than 15 minutes	0.125 h (7.5 min)			
Between 15 and 30 minutes	0.375 h (22.5 min)			
Between 30 minutes and 1 hour	0.75 h (45 min)			
Between 1 and 1.5 hours	1.25 h			
Between 1.5 and 2 hours	1.75 h			
Between 2 and 3 hours	2.5 h			
Over 3 hours	3.5 h			
**Q5.1.** How many times in the last 4 weeks did you do strenuous sports?		Total time in strenuous sports (h/week) = frequency in strenuous sports (times/week) * duration in strenuous sports (h/time)	7.5 METs	Total LTPA= Walking (h/week) * 3.5 METs + light DIY (h/week) * 2.5 METs + heavy DIY (h/week) * 5.5 METs + strenuous sports (h/week) * 7.5 METs + other exercises (h/week) * 5.5 METs
None (if not selected in question 1)	0 times/week			
Once in the last 4 weeks	0.25 times/week			
2-3 times in the last 4 weeks	0.625 times/week			
Once a week	1 times/week			
2-3 times a week	2.5 times/week			
4-5 times a week	4.5 times/week			
Every day	7 times/week			
**Q5.2.** Each time you did strenuous sports, about how long did you spend doing it?				
None (if not selected in question 1)	0 h			
Less than 15 minutes	0.125 h (7.5 min)			
Between 15 and 30 minutes	0.375 h (22.5 min)			

© 2024 Martinez-Gomez D et al. *JAMA Network Open.*

Between 30 minutes and 1 hour — 0.75 h (45 min)<br>Between 1 and 1.5 hours — 1.25 h<br>Between 1.5 and 2 hours — 1.75 h<br>Between 2 and 3 hours — 2.5 h<br>Over 3 hours — 3.5 h			
**Q6.1.** How many times in the last 4 weeks did you do other exercises such as swimming, cycling, keep fit?<br>None (if not selected in question 1) — 0 times/week<br>Once in the last 4 weeks — 0.25 times/week<br>2-3 times in the last 4 weeks — 0.625 times/week<br>Once a week — 1 times/week<br>2-3 times a week — 2.5 times/week<br>4-5 times a week — 4.5 times/week<br>Every day — 7 times/week	Total time in other exercises (h/week) = frequency in other exercises (times/week) * duration in other exercises (h/time)	4.5 METs	
**Q6.2.** Each time you did other exercises such as swimming, cycling, keep fit, about how long did you spend doing them?<br>None (if not selected in question 1) — 0 h<br>Less than 15 minutes — 0.125 h (7.5 min)<br>Between 15 and 30 minutes — 0.375 h (22.5 min)<br>Between 30 minutes and 1 hour — 0.75 h (45 min)<br>Between 1 and 1.5 hours — 1.25 h<br>Between 1.5 and 2 hours — 1.75 h<br>Between 2 and 3 hours — 2.5 h<br>Over 3 hours — 3.5 h			

Example of calculation: participant with the following responses:

Q1: Walking for pleasure, light DIY, heavy DIY, strenuous sports; Q2.1: Every day; Q2.2: Between 30 minutes and 1 hour; Q3.1: 2-3 times a week; Q3.2: Between 15 and 30 minutes; Q4.1: 2-3 times in the last 4 weeks; Q4.2: Between 1 and 1.5 hours; Q5.1: Once a week; Q5.2: Between 1.5 and 2 hours; Q6.1: None; Q6.2: None.

LTPA = 7 times/week * 0.75 h/time * 3.5 METs + 2.5 times/week * 0.375 h/time * 2.5 METs + 0.625 times/week * 1.25 h/time * 5.5 METs + 1 times/week * 1.75 h/time * 7.5 METs + 0 times/week * 0 h/time * 4.5 METs = 18.375 METs-h/week + 2.343 METs-h/week + 4.297 METs-h/week + 13.125 METs-h/week + 0 METs-h/week

LTPA = 38.141 METs-h/week

Example reference: doi: 10.1111/joim.12987

© 2024 Martinez-Gomez D et al. *JAMA Network Open.*

eTable 4. Description of leisure-time physical activity questionnaires: China Kadoorie Biobank

China Kadoorie Biobank				
**Questions and original response options**	**Recoded response options**	**Intermediate variables calculated**	**Energy cost assigned for harmonization**	**Total leisure-time PA (METs-h/week)**
**Question (Q)1.1.** What is your main type of exercise? (Tick one option only)<br>**Q1.2.** About how many hours per week did you do such exercise in total in leisure time?				
Tai chi/Qigong	Open response (hours/week)	Total time (h/week) in Tai chi/Qigong (if selected)	3.5 METs	Total LTPA (if Tai chi/Qigong selected) = Tai chi/Qigong (h/week) * 3.5 METs
Jogging/aerobic exercise	Open response (hours/week)	Total time (h/week) in jogging/aerobic exercise (if selected)	7.5 METs	Total LTPA (if jogging/aerobic exercise selected) = jogging/aerobic exercise (h/week) * 7.5 METs
Walking	Open response (hours/week)	Total time (h/week) in walking (if selected)	3.5 METs	Total LTPA (if walking selected) = walking (h/week) * 3.5 METs
Swimming	Open response (hours/week)	Total time (h/week) in swimming (if selected)	6.5 METs	Total LTPA (if swimming selected) = swimming (h/week) * 6.5 METs
Ball games	Open response (hours/week)	Total time (h/week) in ball games (if selected)	5.5 METs	Total LTPA (if ball games selected) = ball games (h/week) * 5.5 METs
Other (e.g., mountain climbing)	Open response (hours/week)	Total time (h/week) in other (if selected)	4.5 METs	Total LTPA (if other selected) = other (h/week) * 3.5 METs
**Example of calculation**: participant with the following responses:<br>Q1.1: Jogging/aerobic exercise; Q2.2: 9 h/week<br>LTPA = 5 h/week * 7.5 METs<br>LTPA = 37.5 METs-h/week				
**Example reference**: doi: 10.1016/j.lanwpc.2022.100457				

© 2024 Martinez-Gomez D et al. *JAMA Network Open.*

eTable 5. Description of leisure-time physical activity questionnaires: MJ Taiwan 1997-2016

MJ Taiwan 1997

Questions and original response options	Recoded response options	Intermediate variables calculated	Energy cost assigned for harmonization	Total leisure-time PA (METs-h/week)
**Question (Q)1.** What kind of exercise do you usually do? (Multiple choices)<br>Light exercise: gardening, sweeping the floor, mopping the floor, golf, baseball, light aerobics, dancing (regular), biking (slow speed)<br>Medium exercise: basketball, volleyball, table tennis, badminton, dancing (intensive), swimming (as wished), brisk walking<br>Heavy exercise: jogging (8 kilometers per hour), mountain climbing, climbing the stairs, swimming (freestyle and breaststroke)<br>Intensive exercise: running (12 kilometers per hour), jump rope, rowing, swimming (butterfly), speed skating.				
**Q2.** How much time do you devote to regular exercise?<br>None or less than 1 h/week<br>1-2 h/week<br>2-3 h/week<br>Over 4 h/week	 0.5 h/week<br>1.5 h/week<br>2.5 h/week<br>4.5 h/week	Total time in exercise (h/week)	2.5 METs (if only light exercise was selected in Q1)<br>4.5 METs (if only medium exercise was selected in Q1)<br>6.5 METs (if only heavy exercise was selected in Q1)<br>8.5 METs (if only intensive exercise was selected in Q1)<br><br>Mean of METs values for exercises when more than one were selected in Q1 (e.g., if light and intensive exercise were selected in Q1,<br>METs = [2.5+8.5]/2 = 5.5 METs)	Total LTPA=total time in exercise (h/week) * METs assigned (depending on responses on Q1)

MJ Taiwan 1998-2008

Questions and original response options	Recoded response options	Intermediate variables calculated	Energy cost assigned for harmonization	Total leisure-time PA (METs-h/week)
**Q1.** What kind of exercise do you usually do? (Multiple choices)<br>Light exercise: gardening, sweeping the floor, mopping the floor, golf, baseball, light aerobics, dancing (regular), biking (slow speed)<br>Medium exercise: basketball, volleyball, table tennis, badminton, dancing (intensive), swimming (as wished), brisk walking<br>Heavy exercise: jogging (8 kilometers per hour), mountain climbing, climbing the stairs, swimming (freestyle and breaststroke)<br>Intensive exercise: running (12 kilometers per hour), jump rope, rowing, swimming (butterfly), speed skating.				
**Q2.** How much time do you devote to regular exercise?<br>None or less than 1 h/week<br>1-2 h/week<br>3-4 h/week<br>5-6 h/week<br>Over 7 h/week	 0.5 h/week<br>1.5 h/week<br>3.5 h/week<br>5.5 h/week<br>7.5 h/week	Total time in exercise (h/week)	2.5 METs (if only light exercise was selected in Q1)<br>4.5 METs (if only medium exercise was selected in Q1)<br>6.5 METs (if only heavy exercise was selected in Q1)<br>8.5 METs (if only intensive exercise was selected in Q1)<br><br>Mean of METs values for exercises when more than one were selected in Q1 (e.g., if heavy and intensive exercise were selected in Q1,<br>METs = [6.5+8.5]/2 = 7.5 METs)	Total LTPA=total time in exercise (h/week) * METs assigned (depending on responses on Q1)

© 2024 Martinez-Gomez D et al. *JAMA Network Open.*

eTable 5 (cont.). Description of leisure-time physical activity questionnaires: MJ Taiwan 1997-2016

MJ Taiwan 2009-2012				
**Questions and original response options**	**Recoded response options**	**Intermediate variables calculated**	**Energy cost assigned for harmonization**	**Total leisure-time PA (METs-h/week)**
**Q1.** How often do you exercise during the last two weeks? (one choice; first option)<br>Light exercise: gardening, sweeping the floor, mopping the floor, golf, baseball, light aerobics, dancing (regular), biking (slow speed)<br>Medium exercise: basketball, volleyball, table tennis, badminton, dancing (intensive), swimming (as wished), brisk walking<br>Heavy exercise: jogging (8 kilometers per hour), mountain climbing, climbing the stairs, swimming (freestyle and breaststroke)<br>Intensive exercise: running (12 kilometers per hour), jump rope, rowing, swimming (butterfly), speed skating.				
**Q2.1** How often do you exercise during the last two weeks? (first option)<br>none or rarely<br>once a week<br>once every 2-3 days<br>once a day<br>2-3 times a day	 0 times/week<br>1 times/week<br>2.5 times/week<br>7 times/week<br>17.5 times/week	Total time in exercise 1 (h/week) = frequency in exercise 1 (times/week) * duration in exercise 1 (h/time)	2.5 METs (if light exercise was selected in Q1)<br>4.5 METs (if medium exercise was selected in Q1)<br>6.5 METs (if heavy exercise was selected in Q1)<br>8.5 METs (if intensive exercise was selected in Q1)	LTPA in exercise1= total time in exercise1 (h/week) * METs assigned to exercise 1 (depending on responses on Q1)
**Q2.2** How many hours do you spend on exercise during the last two weeks? (first option)<br><0.5 h/time<br>0.5-1 h/time<br>1-2 h/time<br>over 2 h/time	 0.25 h/time<br>0.75 h/time<br>1.5 h/time<br>2.5 h/time			
**Q3.** How often do you exercise during the last two weeks? (one choice; second option)<br>Light exercise: gardening, sweeping the floor, mopping the floor, golf, baseball, light aerobics, dancing (regular), biking (slow speed)<br>Medium exercise: basketball, volleyball, table tennis, badminton, dancing (intensive), swimming (as wished), brisk walking<br>Heavy exercise: jogging (8 kilometers per hour), mountain climbing, climbing the stairs, swimming (freestyle and breaststroke)<br>Intensive exercise: running (12 kilometers per hour), jump rope, rowing, swimming (butterfly), speed skating.				
**Q4.1** How often do you exercise during the last two weeks? (second option)<br>none or rarely<br>once a week<br>once every 2-3 days<br>once a day<br>2-3 times a day	 0 times/week<br>1 times/week<br>2.5 times/week<br>7 times/week<br>17.5 times/week	Total time in exercise 2 (h/week) = frequency in exercise 2 (times/week) * duration in exercise 2 (h/time)	2.5 METs (if light exercise was selected in Q3)<br>4.5 METs (if medium exercise was selected in Q3)<br>6.5 METs (if heavy exercise was selected in Q3)<br>8.5 METs (if intensive exercise was selected in Q3)	LTPA in exercise2= total time in exercise2 (h/week) * METs assigned to exercise 2 (depending on responses on Q3)<br>Total LTPA=LTPA in exercise1 + LTPA in exercise2
**Q4.2** How many hours do you spend on exercise during the last two weeks? (second option)<br><0.5 h/time<br>0.5-1 h/time<br>1-2 h/time<br>over 2 h/time	 0.25 h/time<br>0.75 h/time<br>1.5 h/time<br>2.5 h/time			

© 2024 Martinez-Gomez D et al. *JAMA Network Open.*

eTable 5 (cont.). Description of leisure-time physical activity questionnaires: MJ Taiwan 1997-2016

MJ Taiwan 2013-2016				
**Questions and original response options**	**Recoded response options**	**Intermediate variables calculated**	**Energy cost assigned for harmonization**	**Total leisure-time PA (METs-h/week)**
**Q1.** How often do you exercise during the last two weeks? (one choice)				
Light exercise: gardening, sweeping the floor, mopping the floor, golf, baseball, light aerobics, dancing (regular), biking (slow speed)				
Medium exercise: basketball, volleyball, table tennis, badminton, dancing (intensive), swimming (as wished), brisk walking				
Heavy exercise: jogging (8 kilometers per hour), mountain climbing, climbing the stairs, swimming (freestyle and breaststroke)				
Intensive exercise: running (12 kilometers per hour), jump rope, rowing, swimming (butterfly), speed skating.				
**Q2.1** How often do you exercise during the last two weeks?<br>none or rarely<br>once a week<br>once every 2-3 days<br>once a day<br>2-3 times a day	 0 times/week<br>1 times/week<br>2.5 times/week<br>7 times/week<br>17.5 times/week	Total time in exercise (h/week) = frequency in exercise (times/week) * duration in exercise (h/time)	2.5 METs (if light exercise was selected in Q1)<br>4.5 METs (if medium exercise was selected in Q1)<br>6.5 METs (if heavy exercise was selected in Q1)<br>8.5 METs (if intensive exercise was selected in Q1)	Total LTPA= total time in exercise (h/week) * METs assigned to exercise (depending on responses on Q1)
**Q2.2** How many hours do you spend on exercise during the last two weeks?<br><0.5 h/time<br>0.5-1 h/time<br>1-2 h/time<br>over 2 h/time	 <br>0.25 h/time<br>0.75 h/time<br>1.5 h/time<br>2.5 h/time			

Example of calculation: participant with the following responses (questionnaire completed in 1997):
Q1: light exercise, medium exercise, and intensive exercise; Q2: over 4 h/week
LTPA = 4.5 h/week * (2.5+4.5+8.5)/3 METs-h/week = 4.5 h/week * 5.17 METs-h/week
LTPA = 23.25 METs-h/week

Example of calculation: participant with the following responses (questionnaire completed in 2000):
Q1: heavy exercise and intensive exercise; Q2: 5-6 h/week
LTPA = 5.5 h/week * (6.5+8.5)/2 METs-h/week = 5.5 h/week * 7.5 METs-h/week
LTPA = 41.25 METs-h/week

Example of calculation: participant with the following responses (questionnaire completed in 2010):
Q1: light exercise; Q2.1: 2-3 times a day; Q2.2: <0.5 h/time; Q3: heavy exercise; Q4.1: once every 2-3 days; Q4.2: 1-2 h/time.
LTPA = (17.5 times/week * 0.25 h/time * 2.5 METs-h/week) + (2.5 times/week * 1.5 h/time * 6.5 METs-h/week) = 10.94 + 24.375 METs-h/week
LTPA = 35.31 METs-h/week

Example of calculation: participant with the following responses (questionnaire completed in 2015):
Q1: heavy exercise; Q2.1: once a day; Q2.2: 0.5 -1 h/time.
LTPA = 7 times/week * 0.75 h/time * 6.5 METs-h/week
LTPA = 34.125 METs-h/week

Example reference: doi: 10.1136/bjsports-2021-104961

© 2024 Martinez-Gomez D et al. *JAMA Network Open.*

eTable 6. Harmonization of the educational level for the pooled analysis

Educational level categories	NHIS 1997-2018	UK Biobank	China Kadoorie Biobank	MJ 1997-2016
Low	• 1-11 grade<br>• 12 grade, no diploma	• None of below	• No formal school<br>• Primary	• Illiteracy<br>• Elementary<br>• Junior high (9 grade)
Middle	• High school graduate<br>• GED or equivalent<br>• Some college, no degree<br>• Vocational	• A levels/AS levels or equivalent<br>• levels/GCSEs or equivalent<br>• CSEs or equivalent<br>• NVQ or HND or HNC or equivalent<br>• Other professional qualifications	• Secondary<br>• High school	• Senior high (12 grade)<br>• Vocational
High	• Associate degree: academic program<br>• Bachelor's degree<br>• Master's degree<br>• Professional school degree<br>• Doctoral degree	• College or university	• Technical school/college<br>• University	• College/university<br>• Graduate school

© 2024 Martinez-Gomez D et al. *JAMA Network Open.*

eTable 7. Harmonization of alcohol consumption for the pooled analysis

Alcohol consumption categories	NHIS 1997-2018	UK Biobank	China Kandoorie Biobank	MJ 1997-2016
None or occasional	• Did not drink in past year<br>• Less than one day per week	• Never<br>• Special occasions<br>• 1-3 times per month	• Never or almost never<br>• Only occasionally<br>• Only at certain seasons<br>• Every month but less than weekly	• None or less than 1 time per week<br>• Had quit/former
Infrequent	• 1 day per week<br>• 2 days per week	• 1-2 times per week	• 1-2 days/week	• 1-2 times per week
Regular	• 3 days per week<br>• 4 days per week<br>• 5 days per week<br>• 6 days per week<br>• 7 days per week	• 3-4 times per week<br>• Daily or almost daily	• 3-5 days/week<br>• Daily or almost every day	• 3-4 times per week<br>• Daily

© 2024 Martinez-Gomez D et al. *JAMA Network Open.*

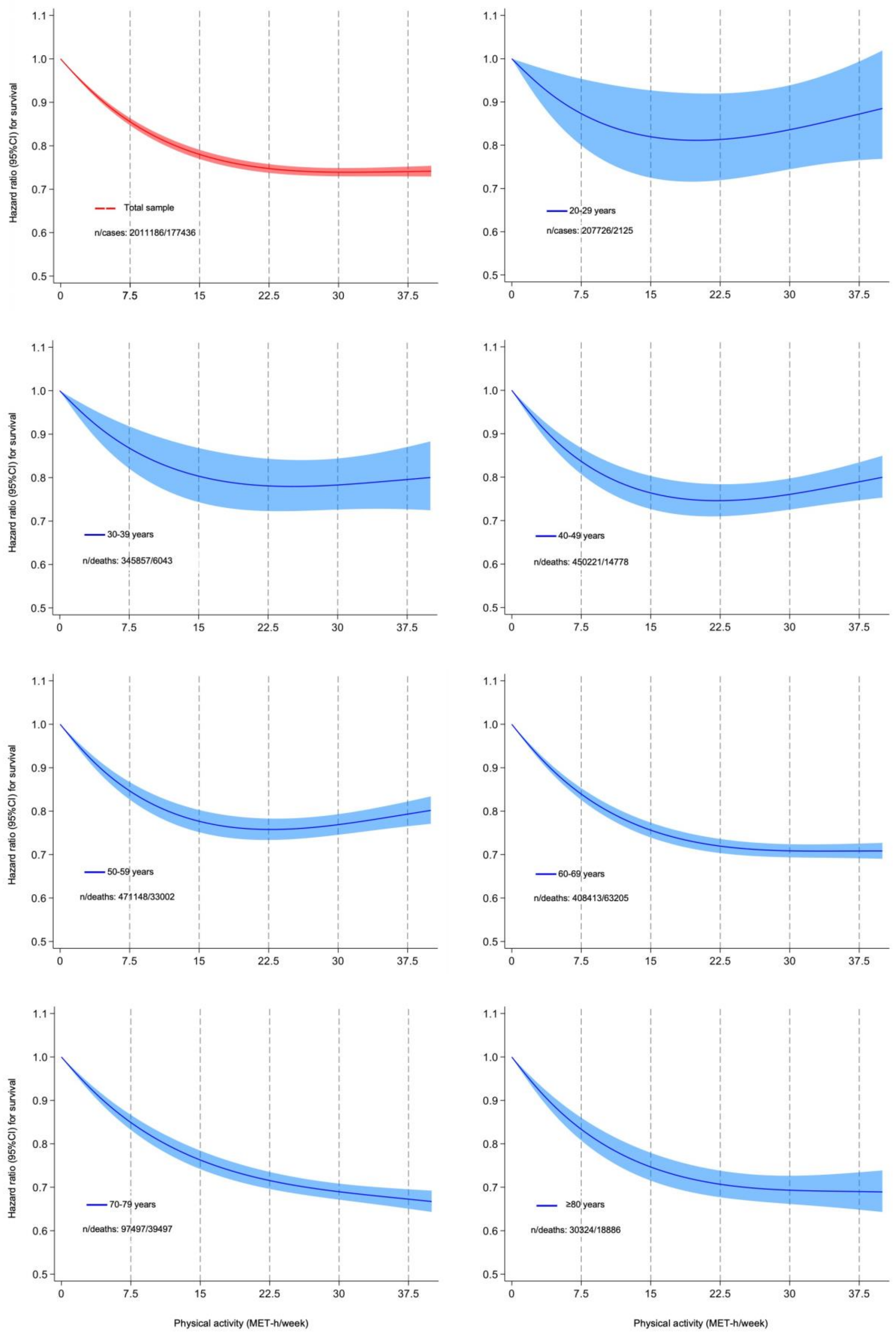

eFigure 1. Dose-response association between physical activity and all-cause mortality in the pooled sample and by age group. Values of 7.5, 15, 22.5, 30 and 37.5 MET-h/week are equivalent to 1, 2, 3, 4, and 5 times the recommended amounts of physical activity, respectively. Analyses were adjusted for study, age, sex, educational level, alcohol drinking, smoking, body mass index, diabetes, hypertension, cardiovascular disease, and cancer.

© 2024 Martinez-Gomez D et al. *JAMA Network Open.*

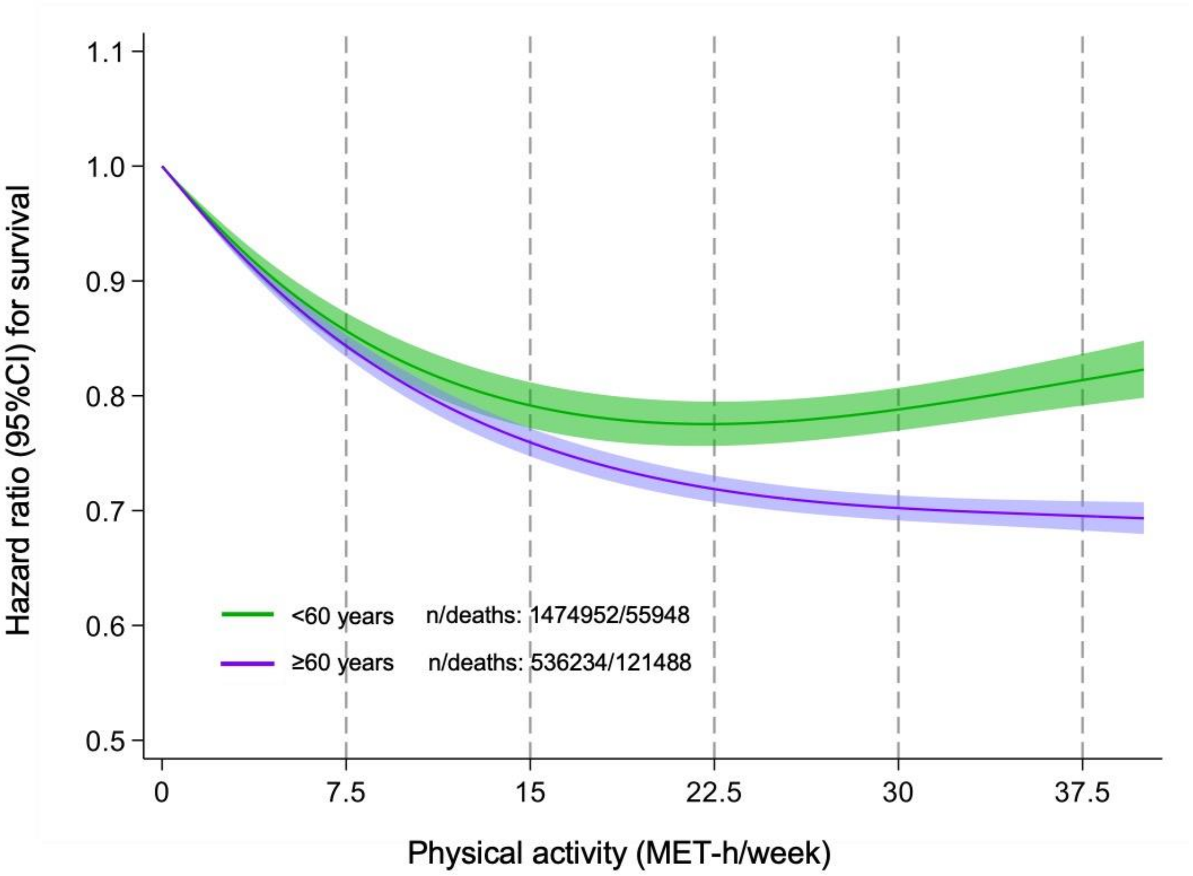

eFigure 2. Dose-response association between physical activity and all-cause mortality in the pooled sample by age group. Values of 7.5, 15, 22.5, 30 and 37.5 MET-h/week are equivalent to 1, 2, 3, 4, and 5 times the recommended amounts of physical activity, respectively. Analyses were adjusted for study, age, sex, educational level, alcohol drinking, smoking, body mass index, diabetes, hypertension, cardiovascular disease, and cancer. P for age-group interaction <0.001.

© 2024 Martinez-Gomez D et al. *JAMA Network Open.*

eTable 8. Risk of mortality associated with meeting the recommended physical activity, stratified by cohort

	Meeting the recommended physical activity HR (95%CI)			
	NHIS	UK Biobank	CK Biobank	MJ
Total sample	0.74 (0.73-0.75)	0.76 (0.74-0.78)	0.75 (0.73-0.77)	0.82 (0.80-0.84)
Total sub-sample*	0.73 (0.72-0.75)	0.76 (0.74-0.78)	0.75 (0.73-0.77)	0.81 (0.79-0.83)
20-29 years	0.81 (0.72-0.91)	-	-	0.94 (0.80-1.11)
30-39 years	0.79 (0.73-0.85)	-	0.85 (0.67-1.08)	0.91 (0.82-1.02)
40-49 years	0.77 (0.73-0.81)	0.75 (0.68-0.82)	0.86 (0.78-0.95)	0.88 (0.81-0.95)
50-59 years	0.73 (0.70-0.76)	0.77 (0.74-0.81)	0.86 (0.81-0.90)	0.83 (0.79-0.88)
60-69 years	0.72 (0.69-0.75)	0.76 (0.73-0.78)	0.73 (0.71-0.76)	0.79 (0.76-0.82)
70-79 years	0.74 (0.71-0.76)	0.64 (0.53-0.78)	0.71 (0.68-0.74)	0.80 (0.76-0.84)
80+ years	0.78 (0.75-0.81)	-	-	0.81 (0.72-0.92)

Analyses were adjusted for age, sex, cardiovascular disease, cancer, and other health factors (including high educational level, not smoking, not regular alcohol drinking, healthy body weight, living without hypertension, and living without diabetes). *The total subsample encompasses only the age groups included in the four cohorts (i.e., 40-79 years). CK: China Kadoorie; NHIS: National Health Interview Survey. UK: United Kingdom. MJ: Mei Jau.

© 2024 Martinez-Gomez D et al. *JAMA Network Open.*

eTable 9. Risk of mortality associated with meeting the recommended physical activity excluding one cohort at a time

	Meeting the recommended physical activity HR (95%CI)			
	Excluding NHIS	Excluding UK Biobank	Excluding CK Biobank	Excluding MJ
Total sample	0.78 (0.77-0.79)	0.79 (0.78-0.80)	0.77 (0.76-0.78)	0.77 (0.76-0.77)
Total sub-sample*	0.77 (0.76-0.78)	0.78 (0.77-0.79)	0.77 (0.76-0.78)	0.76 (0.75-0.77)
20-29 years	0.94 (0.80-1.11)	0.84 (0.76-0.93)	0.84 (0.76-0.93)	0.81 (0.72-0.91)
30-39 years	0.90 (0.82-0.99)	0.83 (0.78-0.88)	0.82 (0.77-0.87)	0.80 (0.74-0.86)
40-49 years	0.83 (0.78-0.87)	0.81 (0.78-0.85)	0.78 (0.75-0.81)	0.78 (0.75-0.81)
50-59 years	0.82 (0.80-0.84)	0.81 (0.78-0.83)	0.77 (0.75-0.79)	0.78 (0.76-0.81)
60-69 years	0.76 (0.74-0.77)	0.75 (0.73-0.76)	0.75 (0.74-0.77)	0.74 (0.73-0.75)
70-79 years	0.74 (0.72-0.76)	0.74 (0.73-0.76)	0.75 (0.73-0.77)	0.73 (0.71-0.75)
80+ years	0.82 (0.72-0.92)	0.78 80.75-0.81)	0.78 (0.75-0.81)	0.78 (0.75-0.81)

Analyses were adjusted for study, age, sex, cardiovascular disease, cancer, and other health factors (including high educational level, not smoking, not regular alcohol drinking, healthy body weight, living without hypertension, and living without diabetes). *The total subsample encompasses only the age groups included in the four cohorts (i.e., 40-79 years). CK: China Kadoorie; NHIS: National Health Interview Survey. UK: United Kingdom. MJ: Mei Jau.

eTable 10. Risk of mortality associated with meeting the recommended physical activity in the pooled four cohorts and by age group stratified by sex and region subgroups.

	Meeting the recommended physical activity Hazard ratio (95% confidence interval)			
	Men	Women	Western cohorts	Asian cohorts
Total sample	0.78 (0.77-0.80)	0.76 (0.75-0.78)	0.74 (0.73-0.75)	0.78 (0.77-0.79)
20-29 years	0.85 (0.75-0.96)	0.81 (0.69-0.95)	0.80 (0.71-0.91)	0.93 (0.79-1.10)
30-39 years	0.82 (0.76-0.89)	0.82 (0.74-0.90)	0.78 (0.72-0.84)	0.90 (0.81-0.99)
40-49 years	0.79 (0.75-0.83)	0.79 (0.75-0.84)	0.75 (0.72-0.79)	0.87 (0.82-0.93)
50-59 years	0.80 (0.77-0.83)	0.77 (0.74-0.80)	0.74 (0.72-0.76)	0.84 (0.81-0.87)
60-69 years	0.74 (0.73-0.76)	0.74 (0.73-0.76)	0.73 (0.72-0.75)	0.75 (0.73-0.77)
70-79 years	0.74 (0.71-0.76)	0.73 (0.71-0.76)	0.73 (0.71-0.75)	0.74 (0.71-0.76)
80+ years	0.76 (0.72-0.80)	0.80 (0.76-0.83)	0.78 (0.75-0.81)	0.81 (0.72-0.92)

Analyses were adjusted for study, age, sex (when appropriate), cardiovascular disease, cancer, and other health factors (including high educational level, not smoking, not regular alcohol drinking, healthy body weight, living without hypertension, and living without diabetes).

© 2024 Martinez-Gomez D et al. *JAMA Network Open.*

eTable 11. Associations between meeting the recommended physical activity and other modifiable health behaviors.

	**Meeting the recommended physical activity, n(%)**		**Age-, sex- and study-adjusted Odds Ratio (95%CI)**
	**No**	**Yes**	
High educational level			
No	967349 (65.6)	506396 (34.4)	
Yes	257155 (47.8)	280286 (52.2)	1.79 (1.77-1.80)
Not smoking			
No	303570 (71.0)	123835 (29.0)	
Yes	920934 (58.2)	662847 (41.8)	1.56 (1.55-1.57)
Not regular alcohol consumption			
No	161420 (44.2)	203952 (55.8)	
Yes	1063084 (64.6)	582730 (35.4)	0.73 (0.73-0.74)
Healthy body weight[a]			
No	285737 (63.1)	167039 (36.89)	
Yes	938767 (60.2)	619643 (39.76)	1.45 (1.44-1.46)
Living without hypertension			
No	216178 (56.0)	170061 (44.0)	
Yes	1008326 (62.1)	616621 (37.9)	1.25 (1.24-1.26)
Living without diabetes			
No	60024 (60.8)	38682 (39.2)	
Yes	1164480 (60.9)	748000 (39.1)	1.38 (1.36-1.40)

[a]CK: China Kadoorie; NHIS: National Health Interview Survey. UK: United Kingdom. MJ: Mei Jau. [a] Doing at least 7.5 MET-h/week. [b] 18.5 ≤ BMI <30.0 kg/m2 in the NHIS and UK Biobank, and 18.5 ≤ BMI <28.0 kg/m2 in the China Kadoorie Biobank and MJ.

eTable 12. Mortality associated with meeting the recommended physical activity in the pooled four cohorts and by age group when excluding some participants and when considering mortality at 5-years follow-up.

	Hazard ratio (95% confidence interval)			
	Excluding former and current smokers[1]	Excluding people with chronic conditions*[2]	Excluding deaths the first 2 years of follow-up[3]	5-year all-cause mortality[4]
Total sample	0.78 (0.76-0.79)	0.79 (0.78-0.80)	0.79 (0.78-0.80)	0.67 (0.66-0.69)
20-29 years	0.80 (0.69-0.91)	0.85 (0.77-0.94)	0.85 (0.77-0.94)	0.82 (0.67-1.01)
30-39 years	0.83 (0.75-0.90)	0.83 (0.78-0.88)	0.83 (0.78-0.88)	0.80 (0.69-0.92)
40-49 years	0.80 (0.76-0.85)	0.79 (0.76-0.83)	0.80 (0.77-0.83)	0.75 (0.69-0.82)
50-59 years	0.79 (0.76-0.82)	0.80 (0.78-0.82)	0.80 (0.78-0.82)	0.75 (0.71-0.79)
60-69 years	0.75 (0.73-0.77)	0.77 (0.75-0.79)	0.76 (0.74-0.77)	0.66 (0.64-0.69)
70-79 years	0.75 (0.73-0.78)	0.75 (0.73-0.77)	0.75 (0.73-0.77)	0.59 (0.56-0.61)
80+ years	0.82 (0.78-0.86)	0.79 (0.76-0.83)	0.80 (0.77-0.83)	0.63 (0.59-0.67)

Analyses were adjusted for study, age, sex, cardiovascular disease (when appropriate), cancer (when appropriate), and other health factors, including high educational level, not smoking (when appropriate), not regular alcohol drinking, healthy body weight, living without hypertension, and living without diabetes. * Indicates cardiovascular disease and cancer. [1]n (total)=2,000,360; [2]n (total)=1,233,617; [3]n (total)=1,820,405; [4]n (total)=2,011,186.

© 2024 Martinez-Gomez D et al. *JAMA Network Open.*
