# Supplementary material for: Physical Activity and All-Cause Mortality by Age in 4 Multinational Megacohorts
Source: JAMA Netw Open. 2024 Nov 21;7(11):e2446802. doi: 10.1001/jamanetworkopen.2024.46802 (PMC11582934; doi:10.1001/jamanetworkopen.2024.46802)
Supplement: Supplement 2. — Data Sharing Statement [file jamanetwopen-e2446802-s002.pdf]

Data Sharing Statement

Martinez-Gomez. Physical Activity and All-Cause Mortality by Age in 4 Multinational Megacohorts. *JAMA Netw Open*. Published November 21, 2024. doi:10.1001/jamanetworkopen.2024.46802

Data
Data available: No

Additional Information
Explanation for why data not available: The four cohorts are available for external researchers.
